# Complete, Fully Automatic Detection and Classification of Benign and Malignant Breast Tumors Based on CT Images Using Artificial Intelligent and Image Processing

**DOI:** 10.3390/jcm12041582

**Published:** 2023-02-16

**Authors:** Chung-Feng Jeffrey Kuo, Hsuan-Yu Chen, Jagadish Barman, Kai-Hsiung Ko, Hsian-He Hsu

**Affiliations:** 1 Department of Materials Science and Engineering, National Taiwan University of Science and Technology, Taipei 106, Taiwan; 2 Department of Radiology, Tri-Service General Hospital, National Defense Medical Center, Taipei 114, Taiwan

**Keywords:** breast tumor, image processing, computer-aided diagnosis, active contour method, sequential forward selection, support vector machine

## Abstract

Breast cancer is the most common type of cancer in women, and early detection is important to significantly reduce its mortality rate. This study introduces a detection and diagnosis system that automatically detects and classifies breast tumors in CT scan images. First, the contours of the chest wall are extracted from computed chest tomography images, and two-dimensional image characteristics and three-dimensional image features, together with the application of active contours without edge and geodesic active contours methods, are used to detect, locate, and circle the tumor. Then, the computer-assisted diagnostic system extracts features, quantifying and classifying benign and malignant breast tumors using a greedy algorithm and a support vector machine. The study used 174 breast tumors for experiment and training and performed cross-validation 10 times (k-fold cross-validation) to evaluate performance of the system. The accuracy, sensitivity, specificity, and positive and negative predictive values of the system were 99.43%, 98.82%, 100%, 100%, and 98.89% respectively. This system supports the rapid extraction and classification of breast tumors as either benign or malignant, helping physicians to improve clinical diagnosis.

## 1. Introduction

Breast cancer is the most common malignant tumor in women around the world [1], causing more than 40,000 deaths every year [2]. As of 1 January 2021, there were more than 2.8 million women with a history of breast cancer in the United States [3]. The survival rate of breast cancer varies by stage at the time of diagnosis. From 2009 to 2015, evidence accumulated that, if tumors in the breast can be found and treated in the initial stage I, the five-year survival rate can be as high as 98%, but when the disease is detected in a late stage, the five-year survival rate is only 27% [1], therefore detection and treatment of breast cancer are very important to increase the survival rate. Most of the studies used mammography for breast screening, which did not provide anatomical features, has low sensitivity, and poor positive predictive value [4,5,6,7,8,9,10]. A good alternative approach, computed tomography (CT), for breast cancer detection, has seen a rapid rise. CT has been studied for breast cancer detection, which can provide anatomical features of breast [4,5]. The studies showed potential anatomical details, which are not appreciated in mammography technology. CT imaging is being used for breast cancer segmentation, which can supply potential assistance and simplifying the workflow for treatment planning [6]. It has potential for breast cancer detection using deep neural networks, which represented better detection results [7]. It can perform well for the detection of breast cancer patient using artificial intelligence and deep learning [8,9,10]. Computer-aided diagnosis (CAD) can play a key role in the early detection of breast cancer and effectively reduce the mortality rate of women with breast cancer [11,12]. Therefore, there are a large number of related CAD systems discussed in the literature and based on these studies, and a system is developed in this study. Lou et al. [13] introduced the assumption that the intensity value from the breast area to the background is a monotonically decreasing function. First, clustering is used to search for the initial boundary. For each initial boundary point, a contour point can be extrapolated and defined by connecting all contour points to compute the breast contour. Keller et al. [14] presented an adaptive fuzzy C-means (FCM) clustering based on an optimal number of clusters derived from the tissue properties of the specific mammogram, followed by generation of a breast parenchymal segmentation through cluster agglomeration using linear discriminant analysis. Iyer et al. [15] applied feature-based fuzzy classification for interpretation of mammograms. FCM clustering is a very powerful method if used with correct settings and parameters adjustment.

Although the above clustering algorithm is accurate, it takes a long time and was judged not to be suitable for the image processing method of this system. Ertas et al. [16] used morphological operations and thresholds for segmentation, but the effect was better when the chest wall contrast was high. The Otsu method [17] searches the threshold value that can distinguish the intra class variance. It computes the histogram and probability of each intensity level, which are then used to determine the possible threshold which can distinguish foreground and background. The advantage of this method is that it automatically separates the foreground from the background without human judgment.

With the above-mentioned binarization threshold segmentation methods plus morphological operations, the efficiency of segmenting the breast ROI is relatively high. Common tumor segmentation methods include the threshold segmentation algorithm [18] and region growth segmentation algorithm [19]. The segmentation algorithm used in the study [18] was the fuzzy algorithm, and this study finds the threshold value, which is used to identify the contour of the ROI. The region growing method [19] was used in ultrasonic imaging because it is difficult to use single threshold values for contouring the ROI. Thus, an iterative method was used to identify the ROI using sliding window size of H x H pixels. When the tumor area has a uniform brightness or smooth texture, this type of segmentation method can obtain accurate segmentation results. However, when there is uneven brightness or rough texture in the tumor, this kind of segmentation method cannot achieve satisfactory performance. Osher et al. [20] proposed the level set function method, projecting the gray-scale image to a higher-dimensional distribution map and using the minimum energy contour line as the contour boundary. Caselles et al. [21] improved the existing geometric active contour model, which can still perform stable boundary detection even when its gradient is subject to large changes. The above methods are all based on the image gradient as the stopping condition of curve evolution, and their ability to detect fuzzy boundaries of a tumor is poor. Chan et al. [22] proposed an active contour without edges (ACWE) method based on regional features, which is mainly based on the active contour method and the traditional level function method, and it is matched with the Mumford-Shah model. This study combines the algorithms of Caselles et al. [21] and Chan et al. [22], using corner detection, to analyze tumor images. When the image gradient is large, Caselles et al. [21] is used for curve evolution based on the image gradient.

CAD systems [23], which integrate diagnosis with computer science, image processing, pattern recognition and artificial intelligence technology, aim to help physicians make diagnostic decisions with the benefit of a “second opinion”. Clinical trials have shown that a CAD system can improve the classification performance of benign and malignant lesions and improve the accuracy of breast cancer detection [24]. Supporting CAD systems and research into the automatic classification of breast lesions have been quite active. Gómez et al. [25] investigated the behavior of co-occurrence statistics combined with gray-scale quantization levels to classify breast lesions on ultrasound (BUS) images. The feature space was ranked using a mutual information technique with minimal-redundancy-maximal-relevance (mRMR) criterion. Yang et al. [26] reported gray-scale invariant features extracted from ultrasound images via multi-resolution ranklet transforms. The linear support vector machine (SVM), when it was run on the resulting gray-level co-occurrence matrix (GLCM)-based texture features, discriminated benign and malignant masses. Wu et al. [27] extracted shape and texture features from breast ultrasound images using a genetic algorithm as a feature selection method and input to a SVM to identify the tumor as benign or malignant. Sadoughi et al. [28] reviewed a total of 18,651 articles discussing artificial intelligence methods for diagnosing breast cancer through image processing from 2007 to 2017. The highest accuracy was achieved with the use of a support vector machine (SVM) for the different types of images (mainly breast ultrasound and mammography).

Most current CAD systems discussed in the computer-aided detection and diagnosis systems literature have focused on mammography, breast ultrasound, and breast magnetic resonance imaging [25,26,27,28,29,30,31], and computed tomography (CT) ones are quite scarce. The CT image has both advantage and disadvantage. Advantages of this study are high diagnostic efficacy in the evaluation of soft tissue tumors, better differentiation of type of tissue, higher reader confidence, etc. The disadvantages are radiation exposure, being expensive, needing a skilled technologist, etc. Even though it has disadvantages, it can provide better tumor characterization using image processing technology. This study appears to be the first to propose a computer-aided detection and diagnosis system for breast tumors targeting CT of the chest. Prior studies, [25,26,27], have shown that the inclusion of breast tumor types and texture features can lead to better classification of benign and malignant tumors. As a result, according to the literature [27,28], when the number of samples is not large, SVM is not only faster, but also more accurate than deep learning. The system in the present study incorporates breast tumor types and texture features combined with feature selection input to a SVM for its classification of benign and malignant tumors.

## 2. Materials and Methods

### 2.1. Breast Tumors

**Figure 1:**
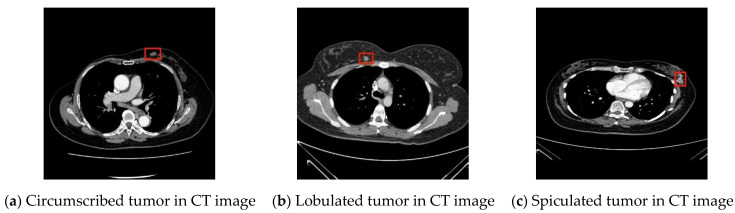
Types of breast cancers.

In this study, breast tumor images were divided into three types according to features of their margins [29,30,31], namely, circumscribed tumors, lobulated tumors, and spiculated tumors, as shown in the Figure 1.

### 2.2. Research Samples and Sample Acceptance Conditions

This study examined the medical records of patients who had undergone chest CT scans at Tri-Service General Hospital between 2010 and 2019. The patients who satisfied conditions for inclusion in the study were: (1) 20 years old or above, (2) had breast tumors found during chest CT examination and were not receiving any chemical or radiotherapy treatment at the time, and (3) had CT images showing more than three tumors or other objects of interest. In the study, a total of 174 patients’ CT image samples was included, of which 89 were benign breast tumor samples and 85 were malignant breast tumor samples. Amongst them, 32 had size of <1 cm and 142 had size of ≥1 cm, as shown in Table 1. The molecular subtypes of malignant tumor are 30 of luminal A, 34 of luminal B, 10 of triple negative, 7 of HER2+, and 4 of unknown. The slice thickness of all images was 1 mm, and the pixel pitch was between 0.5468 and 0.8789 mm. The image width and height were 512 and 666 pixels, respectively.

### 2.3. Image Pre-Processing

The following image processing technique has been used to process the image before starting the analysis. The bilinear interpolation method uses the pixel positions of the four nearest points to a given position P to estimate the pixel value. The adaptive histogram equalization (AHE) method [32] is used to improve the contrast of images produced by breast tumor segmentation. For binarization, Otsu’s method [17] is used to find an optimal threshold to maximize the variance between groups and minimize the variance within groups of the grayscale value characteristics of the image. 

### 2.4. Morphology

Morphology is used to process and analyze the shape of the image [33]. The binarized image produced by the Otsu method may have image defects, such as holes or disconnections. The erosion and dilation, opening and closing, and connected component labeling region filling of morphology methods are used for further processing. 

### 2.5. Contour Extraction Description

The results of contour extraction not only affect the quality of the quantified features extracted from the tumors. They also directly affect the accuracy of the subsequent classification into benign and malignant. The active contour without edges (ACWE) and the geodesic active contour (GAC) methods are used in this system for best results.

#### 2.5.1. ACWE

The level set method based on the area feature of ACWE [34] is used for tumor boundary extraction. The method combines the active contour model [35] and classical level set method with Mumford-Shah segmentation techniques [36], using local region information of the image as the basis for curve evolution. It can detect objects whose boundaries are not necessarily defined by gradient or are not very smooth, which classical active contour models are unable to cope. Because the level set method requires reinitialization, this study follows Li et al. [37] in setting the signed distance function equal to 1, so ACWE is unnecessary on re-initialization, reducing computing time significantly.

#### 2.5.2. GAC

The study also used the gradient feature geodesic level set function method (geodesic active contour, GAC) [38] for tumor boundary extraction. The GAC model incorporates the level set in the active contour method (ACM) model. It takes the gradient of the image as the driving force in achieving convergence at the maximum gradient of the image. This solves the problem of the traditional ACM not being able to handle topological changes in the deformation process. However, the effect is still not ideal when dealing with blurry or textured images.

#### 2.5.3. Shi-Tomasi Corner Detection 

An efficient feature detection algorithm is a crucial task in a computer vision system. Harris corner detection [39] determines whether an image window shows a corner by the degree of change in the window in various directions. The execution of this algorithm is also very fast as compared to the Harris one [39]. This study adopted the Shi-Tomasi corner detector algorithm because it works better than other corner detector algorithms.

### 2.6. Centroid Difference

This method calculates the centroids of the previous object image, the current object image, and the following object image. If the centroid distances are large, the position of the object in the contiguous image slices is changing greatly, indicating that the object may be a cylindrical blood vessel.

$$D=\sqrt{{\left(W_{1}\right)}^{2}-{\left(W_{2}\right)}^{2}}+\sqrt{{\left(W_{2}\right)}^{2}-{\left(W_{3}\right)}^{2}}$$

where D is the centroid difference, and $W_{1}$, $W_{2}$, and $W_{3}$ are the centroids of the front image, the current image, and the back image, respectively.

### 2.7. Greedy Search Algorithm-Sequence forward Selection [40]

The goal of feature selection is to select the best feature subset from the original features so that the classification accuracy can reach a maximum. Suppose we have a set of features $V=\left\{v_{1},v_{2},...,v_{d}\right\}$, and a classification accuracy function, J(V), obtained through a classifier. The goal of feature selection is to find a subset S of V with the best discriminative ability, such that J(S)≥J(T), where T is any subset formed from V. 

### 2.8. Support Vector Machine (SVM)

**Figure 2:**
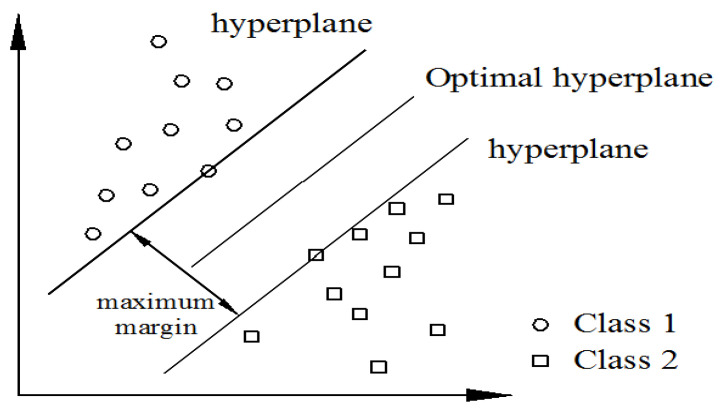
Schematic diagram of support vector machine.

In this study, a SVM was used to classify benign and malignant breast tumors. The basic idea of this supervised machine learning model is to find a hyperplane that maximizes the boundary region between two categories so that they can be perfectly separated. SVMs perform very well in dual classification tasks [41]. Figure 2 shows classification of the region by calculating optimal hyperplane.

### 2.9. The k-Fold Cross-Validation (KCV)

The KCV procedure consists in splitting a dataset into k non-overlapping groups. Then, iteratively, each of the k groups is given the opportunity to act as a held-back test set, while all other k-1 groups collectively are used as a training dataset. A total of k models is thus adapted and evaluated on the k hold-out test sets. The mean performance is then presented. This study uses k-fold (k = 10) cross-validations [42,43] to evaluate its models, because when k = 10, it has the least bias on the machine learning model.

## 3. Results

In this study, image processing technology and SVM were applied in development of a system which can quickly and automatically detect, locate, circle, and diagnose breast tumors in chest CT images, as well as provide read-outs of quantitative data. All the results were verified and ensured by the expertise of a radiologist.

**Figure 3:**
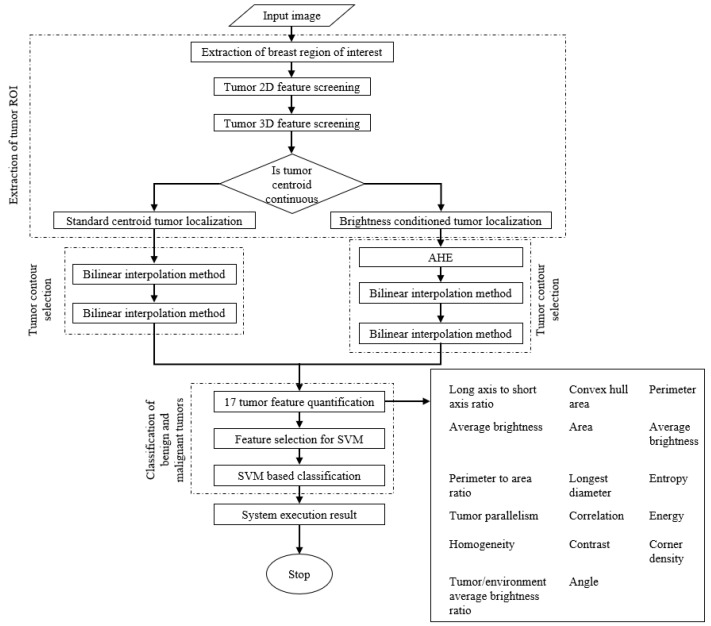
Flowchart of the research process.

A flowchart showing the stages in system processing is presented in Figure 3.

### 3.1. Extraction of Breast Region of Interest 

Since the detected breast tumor is inside the breast, the system first extracts the breast region of interest (ROI) to facilitate subsequent breast tumor screening.

#### 3.1.1. Chest Wall Contour Extraction

**Figure 4:**
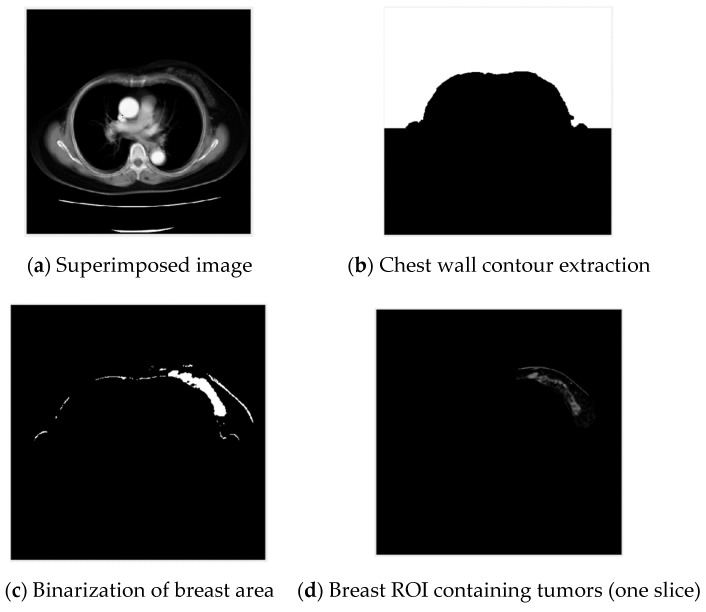
Chest wall contour extraction of CT images.

In order to quickly extract the contour of the chest wall, the entire set of input images are superimposed on each other. One set of images as an example is shown in Figure 4a. Then, the superimposed images are binarized with the Otsu method, as shown in Figure 4b.

#### 3.1.2. Breast Area Distribution Analysis

After the chest wall contour is extracted to obtain the breast ROI, the resulting mask is multiplied back on the image group from which it came. Otsu’s method is used to find possible regions that include breast tissue and tumors, as shown in Figure 4c. Having extracted the breast ROI, the study can now provide information about the breast tumors contained in it. Example breast ROI-containing tumors are shown in Figure 4d.

### 3.2. Extraction of Tumor Region of Interest

Using the two-dimensional features of breast tumors and the continuous features in the entire set of slices, the breast tumor ROI is further extracted.

#### Centroid Approximation-Standard Centroid Tumor Localization

**Figure 5:**
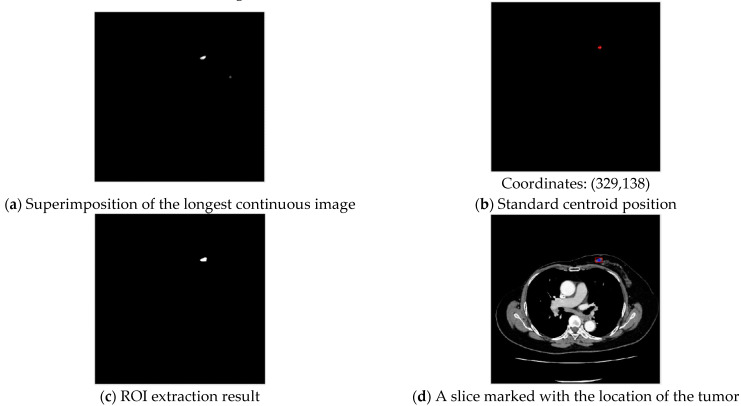
Centroid approximation-Standard centroid tumor localization.

Tumor extraction after longest continuous slice sequence superimposition is shown in Figure 5a. 

The continuous parts of a tumor must be the brightest after superimposition. With Otsu-method binarization, this allows them to be distinguished from other objects that are not tumors, and a standard centroid position can be obtained, with results shown in Figure 5b. With standard centroid positioning, if the distance between each object and the standard centroid location is greater than five pixels, it is filtered out. The system located the example circumscribed tumor in contiguous slices, and the centroid position was (329,138). This completes tumor ROI extraction, with example results displayed on one slice in Figure 5c.

### 3.3. Tumor Contour Selection

#### 3.3.1. Stable Centroid—Breast Tumor Circle Selection

**Figure 6:**
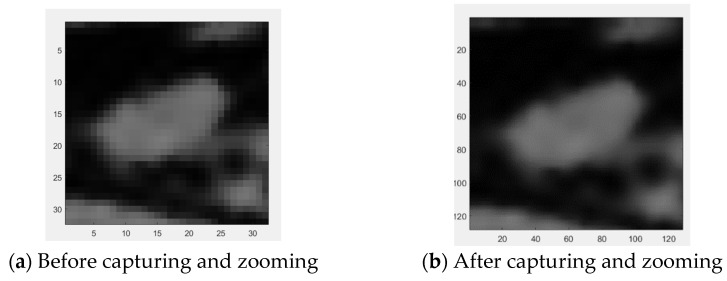
Bilinear interpolation enlargement after acquisition of circumscribed tumor (one slice).

In order to ensure a clear coverage of the tumor within the range of acquisition, the longest diameter of the tumor ROI plus 10 pixels is used as range. With bilinear interpolation, the extracted result is enlarged by four times the side length and 16 times the area, with results shown in Figure 6.

**Figure 7:**
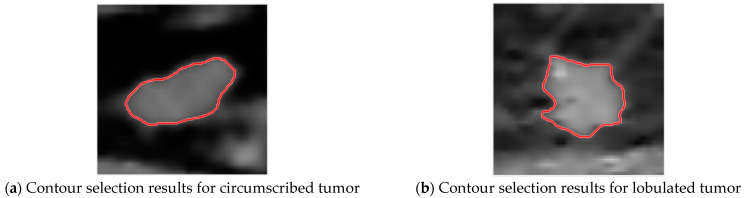
Contour selection of tumors.

Then ACWE is used for tumor contour extraction, with circled results shown in Figure 7.

#### 3.3.2. Excessive Centroid Point Movement—Breast Tumor Circle Selection

Spiculated tumors are tumors with large changes in their centroid positions, but the various parts of the tumor ROI can be extracted and magnified by bilinear interpolation in the same way as with the aforementioned circumscribed and lobulated tumors.

**Figure 8:**
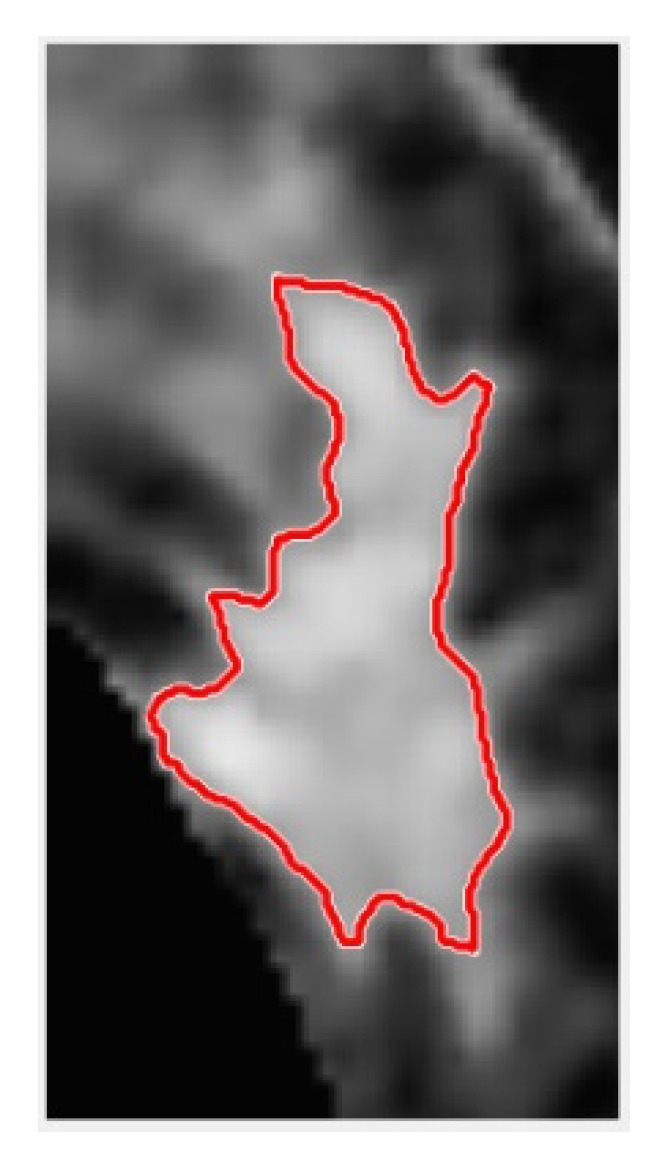
ACWE contour circle selection results for spiculated tumor.

The result of convergence with the region-based active contour ACWE method is shown in Figure 8.

### 3.4. Tumor Feature quAntification 

The tumor feature quantification is conducted based on the features described here as follows: circumference, longest diameter, long axis to short axis ratio, area, perimeter to area ratio, average tumor brightness, average brightness of tumor environment, the ratio of the average brightness of the tumor to the average brightness of the environment, angle, the degree of tumor parallelism, entropy, contrast, correlation, energy, homogeneity of tumor texture, convex hull area, and corner density. 

### 3.5. Classification of Benign and Malignant Tumors

#### 3.5.1. Feature Selection

**Figure 9:**
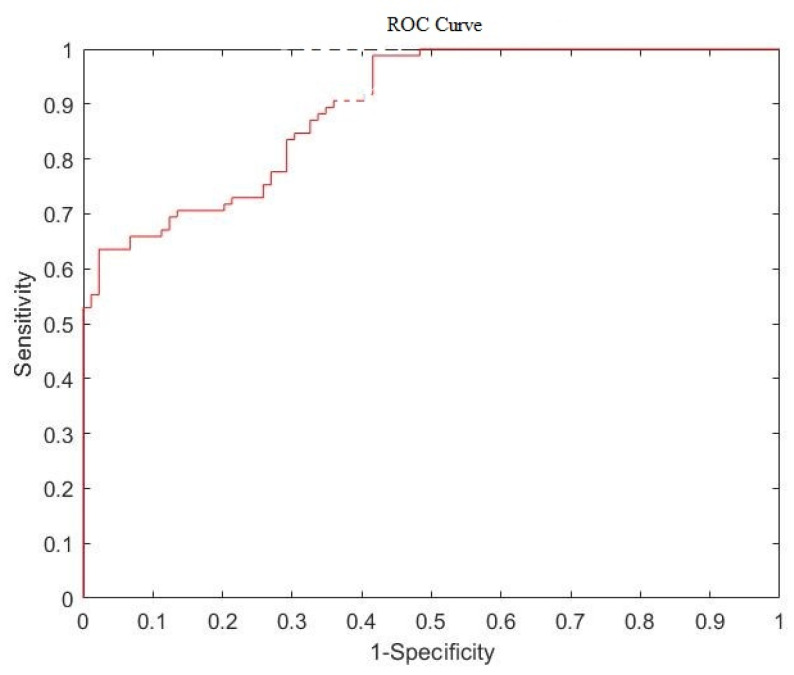
The ROC result of average tumor brightness.

Area under the curve (AUC) of the receiver operating characteristic (ROC) [44,45,46] is determined to measure the importance of the role each feature plays in the identification of tumors as benign or malignant. The ROC result for average tumor brightness is shown in Figure 9, and the corresponding AUC value is 0.8927.

The ROC and AUC of average brightness and the other 16 features were calculated and ranked using the greedy algorithm, as shown in Table 2.

#### 3.5.2. System Effectiveness Evaluation

In the study, positives were defined as malignant tumors, and negatives were defined as benign tumors. The confusion matrix contains information about actual classification and prediction model classification, allowing evaluation of the difference between the detection (prediction) result and the actual situation. The parameters TP, TN, FN, and FP in the confusion matrix are described in Table 3.

The performance of the classifier can be evaluated using standard performance indicators. This study first uses accuracy to find the best SVM classifier model, and then sensitivity, specificity, AUC of ROC, positive predictive value, and negative predictive value for further analysis of the performance of the selected classifier model are used. The six evaluation methods, accuracy, sensitivity, specificity, receiver operating curve (ROC), positive predictive, and negative predictive value, are used to evaluate performance of the study.

#### 3.5.3. Selection of Best SVM Predictive Classifier Model

The classification accuracy of the 17 SVM prediction classifier models obtained from the 17 feature subsets predicting tumors in the test set is shown in Table 4, allowing selection of the best SVM prediction classifier model according to its accuracy.

Model_15 uses a smaller feature set to obtain the highest classification accuracy, meaning it is the best SVM predictive classifier model selected by the system. The study ran cross-validation 10 times [42,43] to analyze the performance of model_15. These features are average brightness, convex hull area, perimeter, average brightness, area, long axis to short axis ratio, perimeter to area ratio, longest diameter, texture (entropy), texture (contrast), tumor texture (correlation), texture (energy), texture (homogeneity), tumor parallelism, corner density, and their corresponding AUC values mentioned in Table 2. The results are shown in Table 5. 

### 3.6. System Execution Result Evaluation

**Figure 10:**
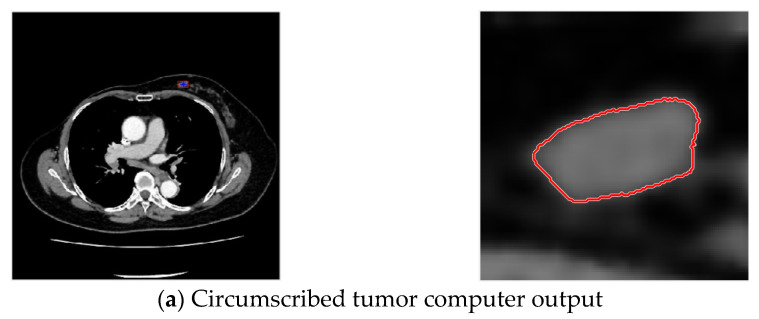

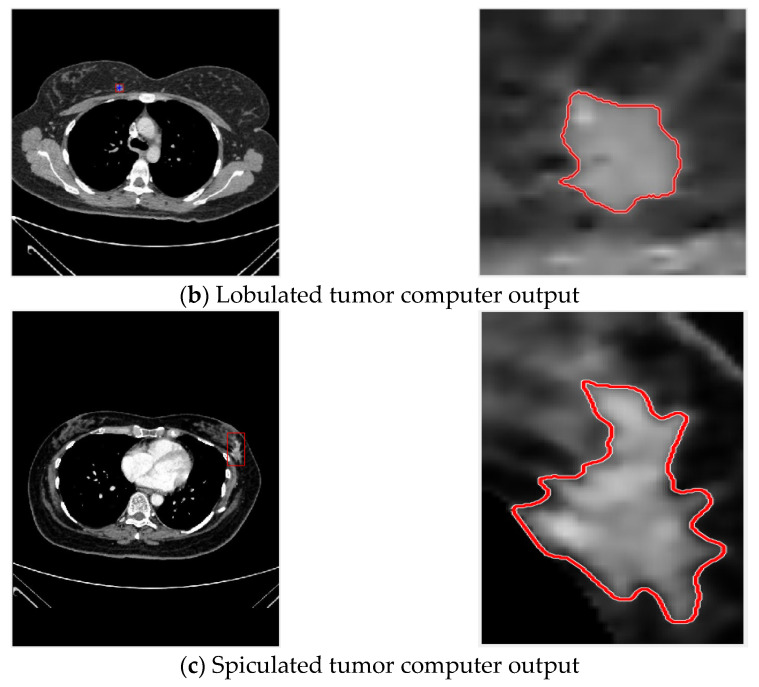
Example tumor computer output.

As for sensitivity for the different tumor types, for circumscribed tumors it was 97.96% (48/49), for lobulated tumors it was 98.89% (89/90), and for spiculated tumors it was 88.57% (31/35). Additionally, the overall sensitivity was 96.55% (168/174). Ten-fold cross-validation was used to evaluate the ability of the classifier to classify benign and malignant tumors. As can be seen from Table 5, accuracy of the trained SVM classifier was 99.43%, AUC value was 0.9941, sensitivity was 98.82%, specificity was 100%, positive predictive value was 100%, and negative predictive value was 98.89%, indicating that the system developed by the study can effectively distinguish benign and malignant tumors. The confidence intervals of AUC and accuracy are calculated at 95% and 99% confidence level, respectively. Regarding the 95% confidence level for the AUC and accuracy, the confidence interval is 0.23 and 0.16, respectively. Regarding © 99% confidence level for the AUC and accuracy, the confidence interval is 0.30 and 0.21, respectively. The system’s result has been shown in the Figure 10.

## 4. Discussion

The breast tumor automatic detection system introduced in this study includes breast ROI extraction, tumor ROI extraction, and tumor circle selection. In order to effectively separate breast tumors and breast tissue, adaptive histogram equalization (AHE) [32] is used to improve breast tumor contrast. AHE obtains good results for images with local areas containing low-contrast bright or dark objects. AHE redistributes the brightness values of the image by calculating a histogram for each separate area of the image. It is more suitable for improving local contrasts and enhancing edge information and was of significant benefit to the subsequent segmentation of breast tumors in this study.

Breast ROI mask is multiplied back on the full set of original images, with bones and other high-brightness parts in the grayscale image filtered out, the high-brightness threshold obtained by the Otsu method allows binarization of each slice. The bright areas displayed in each slice are areas where the breast is very dense, and a tumor is highly likely to exist. Then, the whole set of processed slices are superimposed to find the areas where tumors appear. After that, the study quantified the areas of the dense breast region and the suspected tumor, expressing the area of the suspected tumor as a percentage of the dense breast region to determine the final breast ROI.

**Figure 11:**
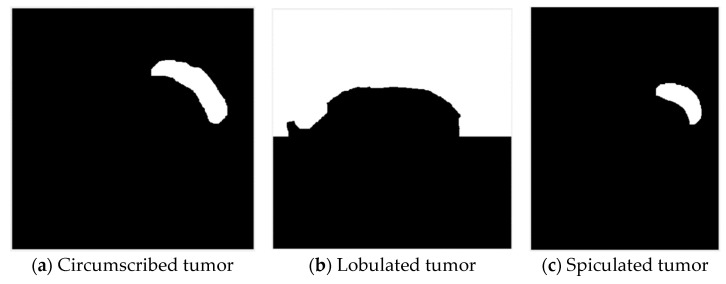
The final breast ROI corresponding to three different tumor image analyses.

In the actual implementation, a standard of five times the area of the suspected tumor is used, and the final breast ROI is selected if the coverage is greater than that. If not, the left and right dense breast regions are both regarded as the final breast ROI. In the example, the coverage is 8.0324 times the area of the suspected tumor. With this analysis, the final breast ROI for the example circumscribed tumor, lobulated tumor, and spiculated tumor are obtained, as shown in Figure 11.

**Figure 12:**
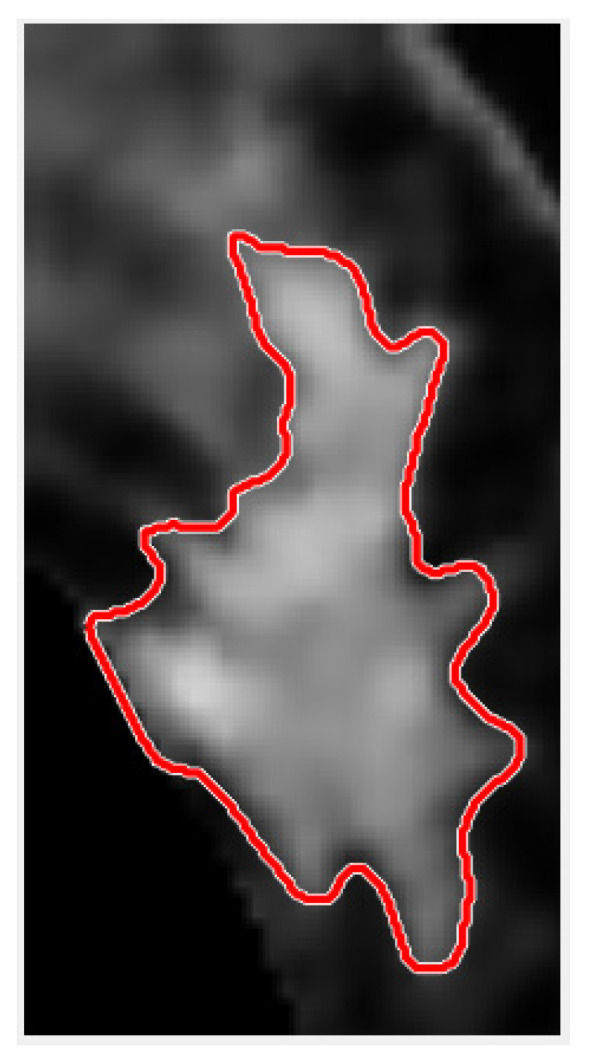
Contour selection result for the spiculated tumor.

In tumor circling, in order to save ACWE iteration convergence time, morphology, threshold segmentation, and centroid positioning are used to automatically obtain an initial contour for the enlarged tumor ROI. This initial version is applied as ACWE input. ACWE can accurately circle tumors in tumor images with blurred borders, but initialization is time-consuming. The results for GAC convergence are opposite to ACWE. GAC results are not ideal when borders are blurred, but the effect is good when there is more detail and large gradients. Therefore, the system dilates the result and then converges it to obtain the final tumor circle, which is displayed on the original image as shown in Figure 12.

Past studies of breast tumor detection by chest CT, and, in particular, Hussain [47], Bach [48], and Poyraz [49], have explored the importance of breast lesions accidentally found in chest CT and the characteristics of malignant and benign breast lesions. Kuo [50] and Caballo [51] only discussed the possibility of automatic segmentation of breast tumors in chest CT. Therefore, this research represents a significant advance with the development of a comprehensive CAD system that provides automatic detection and segmentation of breast tumors, quantitative analysis of features, and a tumor prediction model trained using the quantified characteristics of malignant and benign breast lesions.

In order to establish the competitiveness, and indeed superiority, of the chest CT CAD system developed in this research, Table 6 summarizes the approach and performance of other CAD systems developed in recent years based on mammography, breast ultrasound, and breast magnetic resonance imaging.

In recent years, in the breast tumor CAD system literature, it can be seen that most studies have been based on mammography, breast ultrasound, or breast magnetic resonance imaging, but not chest computer tomography. Usually, they directly rely on tumor extraction features in the image for classification and diagnosis, and they do not mention or ignore the problem of how to automatically detect tumors from a large number of images. From Table 6, it can be seen that the accuracy of CAD systems that only consider texture or morphological features is lower. Therefore, the features studied in this research included size, brightness, angle, texture, border, and other features based on the breast tumor characteristics of chest CT images. In distinction to other CAD systems, this CAD system not only automatically detects breast tumors without manually marking them, but it also achieves the highest classification accuracy of 99.43%.

## 5. Conclusions

This study developed a computer-aided detection system, which extracts breast ROI and incorporates it to extract tumor contours. To cope with the large individual differences in breast tumors and the easy confusion with breast tissue, an algorithm based on Shi-Tomasi corner detection combining the advantages of ACWE and GAC in adaptive extraction of different breast tumor contour types was used. To classify the tumor, the study used the quantification of the features of detected breast tumors and the training of a SVM model to assign tumors to benign or malignant categories. The study used the area under the curve of the receiver’s operating characteristic curve to measure the ability of each feature to distinguish benign from malignant tumors. It found that the highest AUC value of 0.8927 was achieved by average tumor brightness and that it was the best single feature for this task. The degree of tumor parallelism and tumor corner density were considered, and the system provided the best performance, with an accuracy of 99.43% and a sensitivity of 98.82%. This computer-assisted breast tumor diagnosis system not only provides physicians with objective quantitative information about breast tumors, but also ranks the information according to its ability to differentiate malignant from benign tumors, allowing physicians to focus more on the significant information in their diagnoses. Even more helpfully, it also provides a second opinion with an accuracy of 99.43% to assist them.

There were several limitations to our study. First, our study was retrospective in design and introduced selection bias by restricting the entry of patients undergoing CT examination, which was not representative of the general population. Second, the number of lesions (*n* = 174) included in our study was relatively small. A randomized multicenter trial with a larger sample size is needed to determine the relationship between CT enhancement patterns and malignancy. Third, not all patients had histopathological results. Nonetheless, we believed that the imaging acquisition criteria are sufficient to assume the benign nature of breast lesions. The future study can be focused on the uses of deep learning to detect and identify the breast tumors in a wide variety of CT images.

## Figures and Tables

**Table 1:** Size, pathology, and molecular characteristics of the breast lesions.

Lesion Types	Types	Number	Total Number
Benign	Fibroadenoma	69	89
	Fibrocystic change	5	
	Cyst	5	
	Breast abscess	2	
	Intraductal papilloma	3	
	Fibrotic lesion	2	
	Phyllodes tumor	2	
	Intramammary lymph node	1	
Malignant	Invasive ductal carcinoma	48	85
	Invasive lobular carcinoma	10	
	Mixed ductal and lobular carcinoma	9	
	Ductal carcinoma in situ	13	
	Colloid carcinoma	5	
Molecular subtypes of malignant lesion		Total Number	Percentage (%)
Luminal A		28	33
Luminal B		33	39
Triple negative		12	14
HER2+		9	11
Unknown		3	3
Lesion size		Total Number	
<1 cm		32	
>1 cm		142	

**Table 2:** AUC value calculation and ranking of 17 features.

Tumor Feature No.	AUC Value
1. Average brightness	0.8927
2. Convex hull area	0.7764
3. Perimeter	0.75
4. Average brightness	0.7293
5. Area	0.7252
6. Long axis to short axis ratio	0.6906
7. Perimeter to area ratio	0.6875
8. Longest diameter	0.6843
9. Texture (entropy)	0.679
10. Texture (contrast)	0.6537
11. Tumor texture (correlation)	0.6502
12. Texture (energy)	0.6499
13. Texture (homogeneity)	0.5916
14. Tumor parallelism	0.5866
15. Corner density	0.5759
16. Tumor/environment average brightness ratio	0.5687
17. Angle	0.5192

**Table 3:** Forecast and actual comparison.

	Prediction	Detected as Benign Tumor (Negatives)	Detected as Malignant Tumor (Positives)
Ground Truth			
Benign tumor (Negatives)		True Negatives (TN)	False Positives (FP)
Malignant tumor (Positives)		False Negatives (FN)	True Positives (TP)

**Table 4:** Model classification accuracy of 17 different test subsets.

AUC Sort Feature Subset	Number of Selected Features	Classification Accuracy
model_1	1	0.7471
model_2	2	0.9425
model_3	3	0.954
model_4	4	0.9655
model_5	5	0.9655
model_6	6	0.954
model_7	7	0.9597
model_8	8	0.9655
model_9	9	0.9713
model_10	10	0.9828
model_11	11	0.977
model_12	12	0.977
model_13	13	0.9713
model_14	14	0.9885
model_15	15	0.9943
model_16	16	0.9943
model_17	17	0.9943

**Table 5:** Model_15 tumor benign and malignant classification result evaluation.

Accuracy	AUC	Sensitivity	Specificity	Positive Predictive Value	Negative Predictive Value
0.9943	0.9941	0.9882	1	1	0.9889

**Table 6:** Comparison of recently developed CAD systems.

	Application	System Approach	Result
This research	Machine learning in chest CT	Image processing, ACWE, GAC, and SVM	(1) Accuracy: 99.43% (2) Sensitivity: 98.82% (3) Specificity: 100%
Wei [52] (2019)	Machine learning in Breast Ultrasound	Morphological features and SVM	(1) Accuracy: 87.32% (2) Sensitivity: 87.04% (3) Specificity: 87.62%
Vijayarajeswari [53] (2019)	Machine learning in Mammography	Hough transform and SVM.	(1) Accuracy: 94.00% (No others)
AL-Dabagh [54] (2017)	Machine learning in Breast MRI	Traditional image processing and SVM	(1) Accuracy: 94.21% (2) Sensitivity: 95.21% (3) Specificity: 93.33%
Fujioka [55] (2019)	Deep learning in Breast Ultrasound	CNN	(1) Accuracy: 92.50% (2) Sensitivity: 95.8% (3) Specificity: 92.50%

## Data Availability

Not applicable.
